# Molecular Dynamics Simulations of Matrix Metalloproteinase 13 and the Analysis of the Specificity Loop and the S1′−Site

**DOI:** 10.3390/ijms241310577

**Published:** 2023-06-24

**Authors:** Jun Yong Choi, Eugene Chung

**Affiliations:** 1 Department of Chemistry and Biochemistry, Queens College, Flushing, NY 11367, USA; 2 Ph.D. Programs in Chemistry and Biochemistry, The Graduate Center of the City University of New York, New York, NY 10016, USA

**Keywords:** MD simulations, matrix metalloproteinase 13, π−CH(Cβ) interactions, S1′−site, specificity loop, selective MMP inhibitors

## Abstract

The specificity loop of Matrix Metalloproteinases (MMPs) is known to regulate recognition of their substrates, and the S1′−site surrounded by the loop is a unique place to address the selectivity of ligands toward each MMP. Molecular dynamics (MD) simulations of apo−MMP−13 and its complex forms with various ligands were conducted to identify the role of the specificity loop for the ligand binding to MMP−13. The MD simulations showed the dual role of T247 as a hydrogen bond donor to the ligand, as well as a contributor to the formation of the van der Waal surface area, with T245 and K249 on the S1′−site. The hydrophobic surface area mediated by T247 blocks the access of water molecules to the S1′−site of MMP−13 and stabilizes the ligand in the site. The F252 residue is flexible in order to search for the optimum location in the S1′−site of the apo−MMP−13, but once a ligand binds to the S1′−site, it can form offset π−π or edge−to−π stacking interactions with the ligand. Lastly, H222 and Y244 provide the offset π−π and π−CH(Cβ) interactions on each side of the phenyl ring of the ligand, and this sandwiched interaction could be critical for the ligand binding to MMP−13.

## 1. Introduction

Matrix metalloproteinases (MMPs) are zinc−dependent endopeptidases, enzymes that break non-terminal peptide bonds, and degrade extracellular matrix (ECM) proteins, bioactive molecules, and multiple substrates of the central nervous system [1]. Due to their roles in diseased conditions such as cancer progression, MMPs have been interesting therapeutic targets for cancer, osteoarthritis, and cardiovascular diseases [2,3]. MMPs also play vital roles in maintaining human health, such as in wound healing, angiogenesis, and autoimmune inflammation [1]. There are 24 MMP family members, of which catalytic domains are highly conserved with ~60% sequence identity [4]. Since each MMP plays its intrinsic roles in physiological and pathological conditions, pan−inhibition of MMPs can lead to harmful effects on healthy tissues and cells and/or limited beneficial effects on the treatment of human diseases [5]. The failure of former clinical trials using the first−generation MMP inhibitors is mainly attributed to the broad−spectrum inhibition of MMPs and other metalloenzymes [5]. Thus, it is necessary to develop selective MMP inhibitors, which could provide a better understanding of the specific functions of individual MMPs and safer options for clinical trials.

The initial MMP inhibitors are hydroxamic acid−based agents, and due to their Zn-chelating property the inhibitors possessing this moiety show high inhibition potency, but poor selectivity against MMPs and other metalloenzymes [6,7]. Recently, inhibitors targeting exo−sites, or secondary binding sites have been developed, resulting in high selectivity to MMP−13 [8,9]. Since the S1′−sites of MMPs are surrounded by the less conserved specificity loop (Ω−loop), the binders of such sites and their analogs, particularly, extended to the Zn−binding site or the unique K140 of MMP−13, display high selectivity to MMP−13 vs. other MMPs [10]. Interestingly, RF616 (compound 32 in reference [11]) shows high selectivity among MMPs, although its oxetane ring is expected to chelate to Zn ion based on the docking model structure [11]. Thus, the S1′-sites of MMPs are interesting pockets to address selectivity among MMPs, and the discovery of their binders is a compelling tactic for the development of selective MMP inhibitors [12].

Few S1′−site binders have been identified by high throughput screening (HTS) rather than rational design or in silico screening [9]. In addition, these hit compounds were successfully advanced to highly selective MMP inhibitors by combining with other known inhibitors or by targeting K140 [8,11,13,14]. However, the current pool of such binders, or the starting agents, is still very narrow due to the limited access to HTS and the very low success rate of HTS for the discovery of S1′−site binders. In addition, our understanding of the dynamics of the specificity loop in the presence or absence of ligands is still low, especially for its relationship to inhibition potency. The lack of comprehension results in few strategies to identify S1′−site binders except for HTS techniques, although such binders could become valuable starting points for the development of selective MMP inhibitors.

Molecular dynamics (MD) simulation is a powerful computational method to perceive the motion of biomolecules at a molecular level, and it has been increasingly applied to the design of ligands [15]. Particularly, relative binding free energy calculation [16], by a non−physical alchemical method [17], has been utilized for the development of more potent inhibitors, results of which showed acceptable correlations to the experimental data [18,19]. Binding free energies for non−Zn chelating pyrimidine dicarboxamide inhibitors complexed with MMP−13 were calculated by MM/GBSA, which showed a good correlation with the experimental values [20]. In addition, molecular modeling studies of MMP−13 inhibitors such as quinazolinone derivatives were conducted by Quantitative Structure Activity Relationship (QSAR), molecular docking, and MD simulations for statistical analysis and inhibitor design [21]. Zn-binding inhibitors of MMP−9 have been identified via deep−learning and MD simulation approaches [22], and a combination of docking, QM/MM, and MD simulations were employed to estimate the binding affinity of MMP−9 ligands [23]. In addition to the analysis of ligand−enzyme interactions, MD simulations have been utilized to study the functions of metal ions and MMPs. For example, multilevel computational methods such as MD, quantum mechanics (QM), and QM/MM were applied to examine the effects of the nature of metal ions on the catalytic center in MMP−1 [24]. The intermolecular interactions and active site conformations of MMP-1 complexed with collagen were studied using combined meta−dynamics, umbrella sampling, and QM/MM calculations [25,26]. MD simulations of MMP−1− triple−helical peptide (THP) showed substantial conformational dynamics in both components [27], as well as the importance of the linker region between the catalytic (CAT) and hemopexin−like (HPX) domains of MMP−1 [28]. The structure−function relationships of MMP−1 toward THP were investigated by MD simulations of mutants [29], and MD simulations revealed a synergistic impact of the catalytic and structural Zn-ions on the structural stability and dynamics of the MMP−1 − THP [30]. Lastly, QM/MM calculations were employed to study hydrolysis mechanisms of activatable cell−penetrating peptides and oligopeptides by MMP−9 and MMP−2 [31,32]. As briefly described, MD simulations and other computational methods have been extensively utilized to study the ligand−enzyme and substrate−enzyme interactions in MMPs.

Here we present MD simulation results of MMP-13 with a series of ligands, which bind to its S1′−site. Particularly, we focus on the structure activity relationship (SAR) and molecular interactions of such ligands in terms of the dynamics of the specificity loop. We chose compounds 2 and 99–110 in reference [13] because they possess different scaffolds, expected to bind to the S1′−site of MMP−13, with a wide range of inhibition potency to MMP−13 (IC_50_ = 2.5—>5000 nM). In addition, these compounds have a common moiety, which binds to the Zn−binding site and tunnel area, making them relatively fixed during the simulation. Thus, we believe that it is an ideal set of ligands with which to study the unique movement of the specificity loop caused by the presence of a different ligand and its relationship to the inhibition of MMP−13. Note: In this article, these ligands are renamed with the prefix “C” such as C2 and C99–C110 to avoid the confusion of using simple numbers.

## 2. Results

### 2.1. MD Simulations of Apo−MMP−13 Show the Partially Flexible Ω−Loop of MMP−13

**Figure 1:**
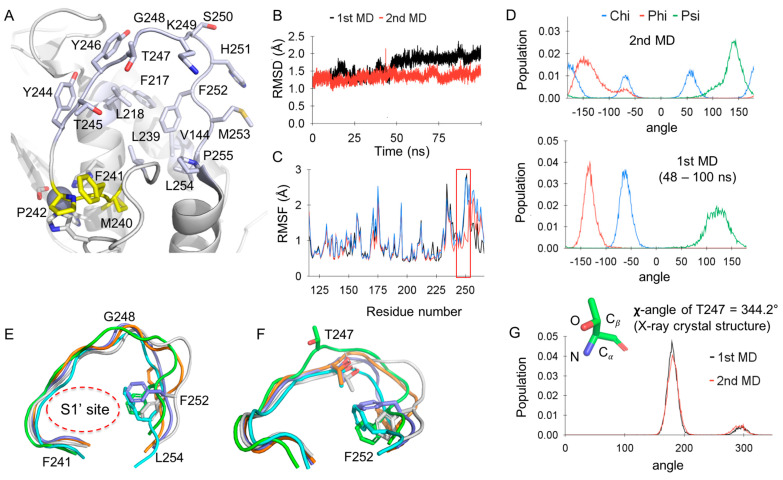
MD simulations of the apo−MMP−13 structure: (**A**) The specificity loop and S1′−site of MMP-13; Residues in the specificity loop and S1′−site are light blue, and residues in the Met−turn are yellow; (**B**) RMSD of MD simulations of apo−MMP-13; (**C**) RMSF of all residues in MD run 1 (blue), run 1 (48–100 ns, red), and run 2 (black); (**D**) Dihedral angle analysis of F252: (top) MD run 2 (0–100 ns) and (bottom) MD run 1 (48–100 ns); (**E**) The specificity loops of MMP−13 from MD simulations; the green loop corresponds to the X-ray crystal structure (PDB code: 4L19).; (**F**) The conformation of T247 and F252 from the MD simulations of MMP-13; the green loop corresponds to the X-ray crystal structure (PDB code: 4L19).; (**G**) The χ−angle histogram of T247 from the MD simulations (20–100 ns) and the χ−angle of T247 in the X-ray co−crystal structure.

The Ω−loop of MMP−13 consists of 11 amino acids (Y^244^TYTGKSHFML^254^), and it is also called the specificity loop since it is less conserved with the distinct combination of amino acids found among MMPs. The loop includes hydrophilic residues of T245, T247, K249, S250, and H251, and glycine (G248) is located in the middle of the loop of MMP−13, accounting for its high flexibility (Figure 1A). In addition, the Ω−loop is connected to the methionine−containing turn (Met−turn), which includes M240, F241, and P242 and forms a hydrophobic pillow for the catalytic zinc ion (Figure 1A) [33,34]. Since the apo−MMP−13 crystal structure was not available, the X-ray co−crystal structure complexed with 1UA (PDB code: 4L19) was used for the system build−up for MD simulations by removing the ligand structure. The AlphaFold structure of apo-MMP−13 was compared with the X-ray co−crystal structure, and their tertiary structures were very similar (Figure S1). In the X-ray co−crystal structure of MMP−13, F252 forms an edge−to−face stacking interaction with F217, and it occupies the hydrophobic surface area composed of F217, L218, L239, L254, P255, and V144 to build the S1′−site (Figure 1A,E). These interactions are commonly detected in all available X-ray co−crystal and NMR structures of MMP−13 except NMR structures (PDB code: 1EUB) [35]. The protein is a homodimer, which contains two conformers (chain A and B), and chain B in the pdb structure was selected for the system buildup. The MCPB.py was used to parameterize the metal sites of MMP−13, such as two Zn ions, two Ca ions, and coordinated amino acids for MD simulations [36,37]. The MMP−13 structure was solvated in a water box with a buffer width of 12 Å. The Amber20 package was used for the MD simulations with three−dimensional periodic boundary conditions at 298.15 K and 1 bar in an NPT ensemble. The standard protocol of Langevin dynamics and Berendsen barostat with a 1 fs time step were applied for the simulations. Two independent MD simulations of apo−MMP−13 were initially conducted for 100 ns.

In the first MD simulation of apo−MMP−13, the RMSD of the enzyme (amide backbone, Cα and Cβ without hydrogen atoms) to the original PDB structure was maintained at 1.9 Å during 48–100 ns (Figure 1B, black scatter plot, slope = 0.0034). The root mean square fluctuation (RMSF) of each residue showed that the loop is highly fluctuated compared to other parts of the enzyme (Figure 1C. red box for Y244−L254). In the second MD simulation of apo−MMP−13, the system converged well with ca. 1.3 Å RMSD (Figure 1B, red scatter plot). However, three distinct conformations of F252 were identified, as indicated by three discrete ranges of χ−angles with similar populations (Figure 1D top). These ranges of the F252 χ−angle can be compared with the single range of its χ−angle from the first simulation, which was highly conserved in the 48–100 ns time period (Figure 1D bottom: dihedral angles from snapshots of 48–100 ns). Thus, the F252 residue was flexible and rotated to search for suitable positions for hydrophobic contacts with F217, L218, L239, L254, P255, and V144 in the S1′−site.

Representative structures having the lowest energies in three different F252 conformations were extracted from simulation trajectories and aligned to the X-ray structure for the comparative analysis. The N−terminal parts (F^241^PIYTYTG^248^) of the specificity loop possess a highly determined conformation, similar to one another, while those of the C−terminal parts (K^249^SHFML^254^) vary according to the orientation of F252 (Figure 1E). The main difference between the X-ray structure (green loop) and the structures from the simulations was the orientation of T247. In the structures from the simulations, the methyl unit of T247 was oriented to the hydrophobic area in the S1′−site, while it is exposed to the solvent area in the X-ray structure (Figure 1F). This conformation of the T247 residue was maintained throughout the 20–100 ns simulation, as shown in the analysis of the χ−angle (Figure 1G; mainly ~170° for the N−Cα−Cβ−O dihedral angle vs. χ = 344.2° for T247 in the X-ray structure). Note: The location of the T247 residue in the chain A is similar to those from simulations, but its methyl unit is still oriented to the solvent area with χ = 66.8°, not to the S1′−site. Therefore, the C−terminal part of the specificity loop is flexible due to the dynamics of the F252 residue and the presence of G248. However, the N−terminal part of the loop could limit its high fluctuation during the simulation, preventing the F252 residue from blocking the S1′ site which a ligand could occupy (Figure 1E; the S1′−site is labeled). In addition, the methyl unit of T247 contributes to the formation of a hydrophobic area in the S1′−site of apo−MMP−13 (Figure 1F).

### 2.2. MD Simulations of MMP−13 Complexed with a Ligand Occupying the S1′−Site

**Figure 2:**
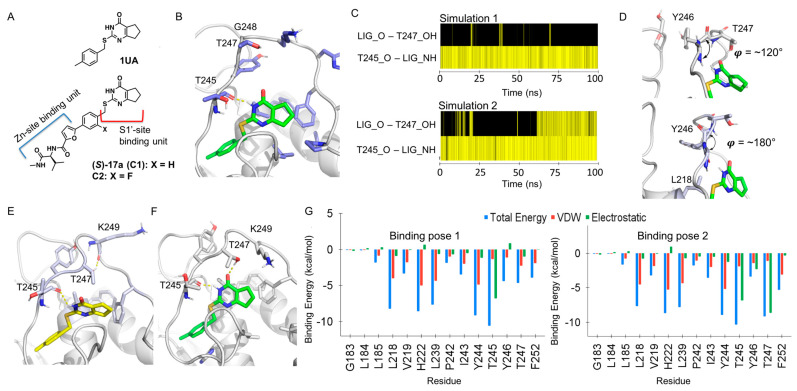
MD simulations of the MMP−13 − 1UA, C1, or C2 complex: (**A**) The structures of 1UA, C1, and C2; (**B**) The open conformation of the specificity loop of MMP−13; (**C**) The H−bond histogram analysis of MMP−13 − 1UA complex; simulation 1 (binding pose 1) and simulation 2 (binding pose 2). The yellow line represents the presence of the H−bond interaction at each time point.; LIG_O and LIG_NH stand for carbonyl oxygen and NH of 1UA, and T245_O and T247_OH for oxygen of the T245 amide backbone and OH of the T247 residue, respectively; (**D**) Two different conformations of T247 depending on the φ angle of Y246; (**E**) The binding pose 1 of 1UA; (**F**) The binding pose 2 of 1UA; (**G**) The per−residue energy decomposition analyses of MMP−13 − 1UA complex; the binding pose 1 (left) and the binding pose 2 (right).

The S1′−site binder (1UA in Figure 2A), possessing 2.4 μM IC_50_, was identified by HTS, and its binding pose was confirmed by the X-ray co−crystal structure (PDB code: 4L19; ligand code: 1UA) [38]. The X-ray co-crystal structure consists of two chains, which show the same binding poses of the ligand (1UA) with slight differences in the Ω-loop. For instance, the T247 residue in chain A is oriented toward the S1′−site where the ligand is located, while that in chain B is oriented toward the solvent accessible area, allowing the ligand to become more exposed to solvent. Since chain A includes an unusual Zn ion to which H251 and glycerol molecules are coordinated, which might be an artifact from crystallization, the chain B structure was used for the system build-up for MD simulations of the MMP−13 − ligand complex. Another ligand, (*S*)−17a (IC_50_ = 9.4 nM) in a reference contains a Zn−site binding unit merged to 1UA (Figure 2A), and it was developed as a highly potent and selective MMP−13 inhibitor by the molecular design and medicinal chemistry efforts [8]. Thus, (*S*)−17a, named as C1 in this study, was docked to MMP−13 for the MD simulations and comparative analysis with 1UA. Although the 250−fold improved inhibition potency of (*S*)−17a (C1) could be interpreted by the structural analysis of the MMP−13 − (*S*)−17a complex (PDB code: 5UWL), we conducted MD simulations to examine if the SAR can be explained quantitively and if unknown interactions can be identified, which could not be detected in the analysis of the X-ray co−crystal structures.

#### 2.2.1. MD Simulations of the MMP−13 − 1UA Complex Showed Two Different Binding Poses of the Ligand, Depending on the Conformations of T247

The MMP−13 − 1UA or MMP−13 − C1 complex in an explicit water box model was simulated for 100 ns, and the binding poses of the ligands and their interactions with MMP−13, particularly in the specificity loop, were analyzed. Two independent simulations of each system were conducted for more samplings (Figure S2; RMSD of two MD simulations). At the beginning of the simulations of the MMP−13 − 1UA complex (up to 60 or 78 ns), the specificity loop of MMP−13 had a wide−open conformation with high flexibility (Figure 2B), though the H−bond interaction between the amide backbone (C=O) of T245 and NH of the pyrimidine−4−one scaffold was retained (Figure 2C, T245_O−LIG_NH). The hydroxyl group of T247 had an H−bond interaction with the carbonyl oxygen (C=O) of the pyrimidin−4−one scaffold, intermittently (Figure 2C, LIG_O−T247_OH). Since T247 is located right next to glycine (G248) in the loop, the conformation of the T247 residue highly depends on that of the Y246 residue. As the Y246 residue faces the neighboring loop (φ = ~120°, C′−N−Cα−C′ dihedral angle), T247 is oriented to the S1′−site to form the H−bond interaction with the ligand (Figure 2D top). However, the Y246 residue forms the hydrophobic interaction with L218, which is perpendicular to the neighboring loop (φ = ~180°, C′−N−Cα−C′ dihedral angle), and T247 is pointing to the solvent accessible area, making the S1′−site more exposed to solvent (Figure 2D bottom and Figure 2B).

After a 60 or 78 ns time period, each independent simulation converged to the unique conformation of the specificity loop. In simulation 1, the methyl group of T247 was involved in the hydrophobic contacts in the S1′−site, while its hydroxyl group formed an intermolecular H−bond interaction with the amide backbone of K249 (Figure 2E). Due to the methyl group of T247 in the S1′−site, the 1UA scaffold was lifted and more exposed to the solvent accessible area, while the van der Waal attractions in the S1′−site were enhanced by the methyl group of T247 (binding pose 1). The MD simulation of this system was extended to 150 ns to search for another possible conformation of T247 and 1UA, but the system was maintained without a significant change in the loop and the ligand.

In simulation 2, another conformation was identified, and the hydroxyl group of T247 formed the H−bond interaction with 1UA from the solvent accessible area, which pushed the scaffold toward the hydrophobic area of the S1′−site (Figure 2F). The methyl groups of T247 and T245 formed the van der Waals attraction, which partially blocked the exposure of the scaffold to solvent. Similarly, the conformation of F252 was frequently changed to find the optimum van der Waals contacts between the scaffold and hydrophobic residues in the S1′−site. In addition, K249, particularly its methylene units, moved to the scaffold to maximize the hydrophobic contacts between the scaffold and residues in the loop and the S1′−site (binding pose 2). This conformation was maintained throughout the 100 ns simulation. Note: During 95–100 ns, F252 was oriented to form the edge−to−face interaction with the scaffold, which made the loop wider and lessened the intermolecular interactions of the enzyme, resulting in the denatured structure.

Since two unique binding models were observed by analyzing two trajectories, 8 additional simulations were conducted using coordinates obtained from the equilibration stages. Thus, 4 copies from 5, 10, 15, and 20 ps NPT equilibration steps of the above MD run 1 and 2 were used for 100 ns simulations. Among these additional simulations, three MD runs converged to the binding pose 1 (run 3, 7, and 9), and the binding pose 2 was also identified from one MD simulation (run 6), as summarized in Table S1. In the other simulation runs, the binding orientations of the ligand were similar to that of pose 2, but the specificity loop mainly had the open conformation, as shown in Figure 2B. Therefore, from the MD simulations of the MMP−13 − 1UA complex (1 μs total), two unique binding poses of the ligand were detected, depending on the conformation of the specificity loop. In addition, various open states of the loop were also observed during the simulations, which might be intermediate or transition states between two binding poses. Two independent MD simulations of the MMP−13 − C1 complex were also conducted, which showed two different conformations of T247 and their interactions with the pyrimidin−4−one scaffold of C1 in the same way as the MMP−13 − 1UA complex.

#### 2.2.2. MM/GBSA Calculations Confirmed the More Stable Conformation (Binding Pose 2)

To determine the binding affinities of two unique binding poses of 1UA, MM/GBSA calculations were performed with 1000 snapshots in the 90–100 ns or 70–90 ns time period of the MD simulation 1 or 2, respectively [39]. The MM/GBSA calculations showed that binding pose 2, having the H−bond interaction with the hydroxyl group of T247 (Figure 2F), was more stable than pose 1 with the van der Waals interaction with the methyl group of T247 (Figure 2E): ΔG_binding_ = −20.6 kcal/mol vs. −13.3 kcal/mol for the pose 2 vs. 1, respectively (Table 1). Furthermore, the binding affinities of poses 1 and 2 in the additional MD runs showed similar ΔG_binding_ values: −20.3 kcal/mol for pose 2 vs. ca. −13.3 kcal/mol (average of run 3, 7, and 9) for pose 1 (Table S1). The ligand in the open conformations of the specificity loop also had a lower binding affinity than pose 2: ΔG_binding_ = −14.0 kcal/mol (average of run 4, 5, 8, and 10) vs. −20.4 kcal/mol (average of run 2 and 6), respectively. Interestingly, the binding affinity without the entropy approximation (ΔG_MM/GBSA_) showed the same trend as ΔG_binding_, which showed ca. 7 kcal/mol difference between pose 1 and 2 in both ΔG_binding_ and ΔG_MM/GBSA_. Therefore, the entropy effect may not be significant for the ligand binding, although the S1′−site is partially encircled by the specificity loop. C1 had the same result as 1UA, resulting in ΔG_binding_ = −22.1 kcal/mol for pose 2 vs. ΔG_binding_ = −16.1 kcal/mol for pose 1. The per-residue energy decomposition analyses (EDA) of the 1UA binding poses showed that T247 had a lower total energy of interaction with pose 2 (−9.2 kcal/mol) than pose 1 (−4.7 kcal/mol) (Figure 2F). In addition, the contribution from the electrostatic energy to its total energy in the binding pose 2 is more significant than that from the van der Waal energy in binding pose 1 (−8.6 kcal/mol vs. −1.0 kcal/mol for binding pose 2 and 1, respectively). F252, which formed van der Waal interactions with hydrophobic residues in the apo−MMP−13, showed such interactions with the ligand, and binding pose 2 had a slightly higher binding affinity by F252: −5.3 kcal/mol vs. −3.9 kcal/mol for the binding pose 2 and 1, respectively in Figure 2G. The π−π stacking interaction between H222 and the 4−methylphenyl ring of the ligand can be easily detected in the X-ray co-crystal structure, which was validated by the MM/GBSA calculation and per−residue energy analysis. Interestingly, both Y244 and H222, particularly their van der Waal energy, had similar contributions to the binding affinity of the ligand to MMP−13. Lastly, L218 and L239, located in the S1′−site, positively contributed to the ligand binding via van der Waals interactions.

The MD simulations and MM/GBSA energy calculations have determined the preferred binding orientations of 1UA and C1. The H−bonding interactions between the hydroxyl group of T247 and the amide backbone of T245 with the pyrimidin−4−one scaffold could be a main contributor to its stabilization in the S1′−site. In addition, the methyl groups of T245 and T247 and the methylene units of K249 are orchestrated to construct the hydrophobic surface, which could shield the scaffold of the ligand from the access of water molecules. F252 frequently rotates to find the optimal position to maximize the hydrophobic contacts with the scaffold and other hydrophobic residues in the S1′−site. However, the contributions of F252 along with L218 and L239 for the ligand binding are high, as shown in the per−residue EDA analysis. The 4−methylphenyl ring of 1UA can be stabilized by π−π stacking and π−CH(Cβ) hydrophobic interactions with H222 and Y244 on both sides of the ring.

#### 2.2.3. The Zn−Site Binder of C1 Positively Contributes to Its Inhibition to MMP−13

**Figure 3:**
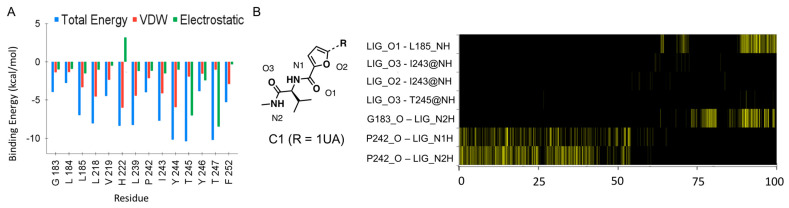
Analysis of MD simulations of MMP−13 − C1 complex: (**A**) The per−residue energy decomposition analyses of MMP−13 − C1 complex; (**B**) The H−bond histogram analysis of MMP−13 − C1 complex; the yellow line represents the presence of the H−bond interaction at each time point. Only the H−bond interactions in the Zn−binding site are present.; LIG_O1, O2, and O3 stand for oxygen of C1, and LIG_N1H and N2H for hydrogen on the amide unit of C1 (left panel). L185_NH/I243_NH/T245_NH and G183_O/P242_O stand for hydrogen and oxygen on the amide backbone of each amino acid, respectively.

The ligand, C1, has additional units merged to 1UA, such as furan, amino acid, and methylamine (the Zn−site binder, Figure 2A), and these units positively contribute to the elevated inhibition against MMP−13. MM/GBSA calculations showed that C1 had enhanced binding affinity to MMP−13, compared to 1UA (ΔG_binding_ = −22.1 kcal/mol vs. −20.6 kcal/mol for C1 vs. 1UA, Table 1), which corresponds to the increased inhibition potency of C1 against MMP−13 in biochemical assays: IC_50′_s (C1 and 1UA) = 9.4 nM and 2400 nM, respectively. The per−residue EDA analysis showed that the hydrophobic interactions between the isopropyl moiety of C1 and L183, L184, L185 and I243 residues were increased (Figure 3A), compared to those in 1UA (Figure 2G). The H−bond interactions between two amide moieties of C1 and main chains of L185, G183, Y244, and P242 were previously described as one of the main components for its elevated inhibition in the X-ray co−crystal structure analysis (Figure 4E in ref [8]). However, the H−bond analysis of MD trajectories showed that these interactions were not substantially detected in the 100 ns MD simulations (Figure 3B), due to the solvation of C1 in the Zn−binding site (Table S2) and water−bridged H−bond interactions with G183 (Table S3). Therefore, the small van der Waals surface area between the Zn−binding unit of C1 and P242/I243 could be easily affected or broken by water molecules, resulting in the highly flexible Zn−binding unit of C1.

### 2.3. A Fluorine Atom on the Phenyl Ring of C1 Improves Its Inhibitory Effect on MMP−13

A ligand, C2, has a fluorine atom at the X−position of the phenyl ring of C1 (Figure 2A), and C2 has a 3.5−fold higher inhibition potency against MMP−13 than C1. Since the X-ray co−crystal structure of the MMP−13 − C2 complex is not available, it is difficult to determine the basis of its slightly improved inhibition against MMP−13 and the exact location of F in the ligand binding site. Thus, MM/GBSA calculation was conducted to determine the location of the fluorine atom on the phenyl ring of C2 in its binding site of MMP−13. The equilibrated coordinate of MMP−13 with the lowest energy from the MD simulations of the MMP−13 − C1 complex was extracted, and two C2 structures were merged to the MMP−13 to build up the systems for simulations: C2A (F is oriented to the Met−turn) and C2B (F is oriented to an alpha−helix). Since the MMP−13 structure was obtained from the 100 ns MD simulation with C1, the new systems with C2 were stabilized within 30 ns simulations, without significant conformational changes. MM/GBSA calculations for the last 10 ns simulations (1000 snapshots) showed similar binding affinities: ΔG_binding_ = −21.3 (4.3) kcal/mol and −20.6 (5.5) kcal/mol for C2A and C2B, respectively.

C1 is 4−fold less potent than C2 in biochemical assays, but it shows a slightly better binding affinity with ΔG_binding_ = −22.1 kcal/mol than C2 (ΔG_binding_ = −21.3 or −20.6 kcal/mol) in MM/GBSA calculations. Since these binding energies are within the error ranges and inconsistent with the experimental data, relative binding free energies by the thermodynamic integration (TI) method were calculated [16]. Briefly, a 3−step protocol was applied for 10 windows (λ) by removing the charge on hydrogen (H28) of C1, changing van der Waals and bonded terms (H28 to F1), and recharging the fluorine (F1) of C2. For each λ, the system was heated for 52 ps of NVT and equilibrated for 35 ps of NPT. Afterward, 4 ns of NPT simulation was performed, and dV/dλ was collected for every 2 ps for each simulation. The TI gradients were integrated by a linear extrapolation to compute ΔG for ligands and complex, and ΔΔGs were calculated by the equation of ΔΔG = ΔG_complex_ − ΔG_ligands_. As shown in the calculated relative binding free energies, ΔΔG_(C1 to C2A)_ = −1.19 kcal/mol and ΔΔG_(C1 to C2B)_ = 2.82 kcal/mol, the C2A conformation showed the better binding affinity to MMP−13 than the C2B conformation, and C1 to C2A transformation increased the binding affinity, while it was reversed in the transformation of C1 to C2B. Thus, according to the relative binding free energy calculation by the TI method, the C2A conformation of which F is pointing to the Met-turn is consistent with the experimental results.

### 2.4. MD Simulations of MMP−13 − Ligands with Distinct Pyrimidin−4−One Scaffolds

**Figure 4:**
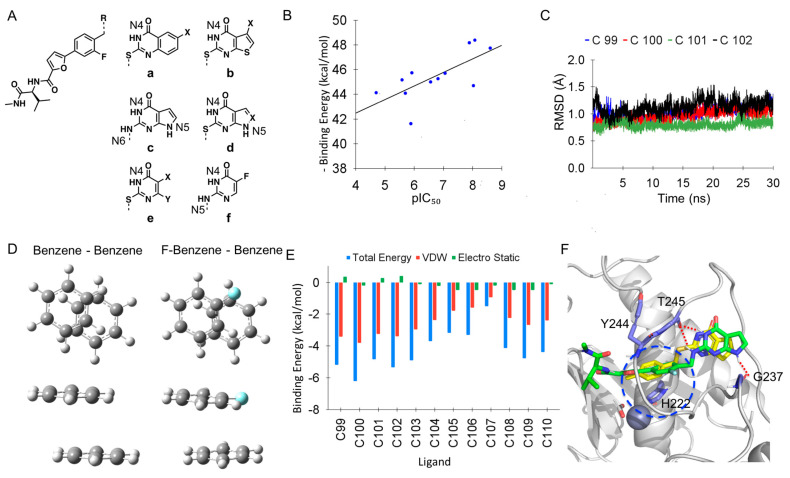
Analysis of MD simulations of MMP−13 complexed with various ligands occupying S1′-site: (**A**) Structures of ligands used for MD simulations; **a**–**f** are scaffolds of ligands, and the complete 2−D structures of ligands are present in Table S4.; (**B**) A data plot of pIC_50_ vs. binding affinities calculated from MM/GBSA; (**C**) RMSD of MD simulations of MMP−13 complexed with C99, C100, C101, and C102; (**D**) The offset π−π stacking interactions of benzene and fluorobenzene obtained from QM calculations; (**E**) The F252 energy decomposition analyses of MMP−13 complexed with C99−C110 via MM/GBSA calculations; (**F**) The shift of C103 (green) in the S1′−site due to additional H−bond interactions.

The ligands (C2, C99−C110) in Figure 4A and Table 2 possess various scaffolds, which are expected to occupy the S1′−site partly formed by the specificity loop, in the same manner as C1 in the X-ray co−crystal structure of MMP−13 [13]. Glide docking studies of these ligands to MMP−13 in the extra precision mode (XP mode) without any constraints also produced the same binding orientations of these ligands to that of (*S*)−17a in the X-ray co−crystal structure (PDB code: 5UWL, Figure S3). Interestingly, although these ligands have scaffolds of reasonable size which are able to be located in the S1′−site without any steric clashes with the target enzyme, their inhibition potencies are within the wide ranges of IC_50′_s: >5000 nM−2.5 nM. Thus, we conducted MD simulations to investigate the binding poses of these scaffolds and their relationship to inhibition potencies (or binding affinities). The equilibrated structure of the MMP−13 − C1 complex from the previous MD simulations was employed to build up the systems for the MD simulations, and C1 was replaced with each ligand structure. The fluorine atom was placed in the same way as C2A, which gave the better binding affinity in the relative free energy calculation. Since the MMP−13 structure was obtained from the prior 100 ns simulation and no significant changes of conformations and potential energies were observed for 30 ns simulations, the trajectories were subjected to analysis, after 30 ns simulations. Three independent MD runs for each ligand were performed. Note: Four MD runs for C106 was conducted.

MM/GBSA calculations were conducted by using the 1000 snapshots from the last 10 ns simulations (20 ns–30 ns time period) for each ligand. Since three independent MD runs were performed, we chose the total energy from three independent MD runs for each ligand, which can be fitted well with pIC_50_ values obtained from the reference [13], and a correlation plot with R^2^ = 0.49 was obtained (Figure 4B). Thus, the MD simulation run selected for the data plotting for each ligand was subjected to further analysis to identify key factors of activity or inactivity of ligands to MMP−13.

#### 2.4.1. MD Simulations of MMP−13 with Ligands Having an Aromatic Ring Occupying the S1′−Site Showed the Parallel and Offset π−π Stacking Interaction with F252

C99, C100, C101, and C102 possess phenyl, fluorophenyl, thiophene, and 3-thiophene rings fused to the pyrimidin−4−one scaffold, and they have similar high inhibition potencies against MMP−13 with a range of 2.5–13 nM IC_50′_s. MD simulations of these ligands complexed with MMP−13 showed that the H−bond interactions between the amide backbone of T245 (T245_O) and NH of the scaffolds (LIG_N4H) were mostly formed with over 90% throughout the simulations (Table 2). The H−bond interactions of the T247 residue (T247_OH) and the carbonyl unit of the scaffolds (LIG_O) were maintained in around 25–60% of the simulations.

The fused rings of C99, C100, C101, and C102, such as phenyl and thiophene rings, formed parallel and offset π−π stacking interactions with the F252 residue during the simulations. L218 is located in the face of the pyrimidin−4−one scaffold, and the flexibility of the N−terminal part of the loop is limited (Figure 1A,E). Thus, the F252 residue could not move closer to the center of the scaffold to form parallel π−π stacking interaction. In the case of C99, the F252 residue rotated to form an edge−to−face stacking interaction with the scaffold. However, significant conformational changes in the F252 residue and the specificity loop were not detected in the simulations (Figure 4C). Thus, the ligands can be stabilized mainly by the H−bond interactions with T245 and T247 and offset π−π stacking interaction with F252 for a high inhibition potency to the target enzyme. In addition, fluorine on the aromatic ring is known to increase the π−π stacking interactions with a benzene ring [40]. The QM calculations of two benzene rings and benzene−fluorobenzene rings with MP2/6−31+G* of the theory and basis sets identified the local minimum structures of the offset π−π stacking of the complexes (Figure 4D). The binding free energies of benzene−benzene, and benzene−fluorobenzene complexes were estimated from the free energy calculations of benzene, fluorobenzene, and their complex forms. The results showed that the benzene−fluorobenzene complex had 0.84 kcal/mol more stable free energy than the benzene−benzene complex: ΔG = 3.85 kcal/mol vs. 4.68 kcal/mol, respectively. Thus, the increased inhibition potency of C100 vs. C99 against MMP−13 can be explained by the more favorable π−π stacking interaction in the scaffold with fluorine. Note: The QM calculation of quinazolin−4−one and benzene rings was conducted, but the benzene ring moved to the middle of the quinazolin−4−one ring to form a more stable π–π stacking complex. However, this complex form is not relevant to the binding pose of C99/C100 due to the limited flexibility of the specificity loop (Figure 1E), and it was not considered in this analysis. Therefore, we used the simplified forms for the interactions between phenylalanine and quinazolin−4−one or 6−fluoroquinazolin−4−one.

C106–C110 do not have any fused ring in the pyrimidin−4−one scaffold, and their inhibition potencies against MMP−13 are substantially decreased, compared to C99–C102. The absence of the π−π stacking interactions with F252 can account for the reduced inhibition of these ligands. During the simulations, the pyrimidin−4−one scaffolds of C106–C110 mainly formed H−bond interactions with the amide backbone of T245 and the T247 residue in a similar way to C99–C102 (Table 2). However, the F252 residue fluctuated, resulting in its low contribution to the binding affinity of the ligand to MMP−13, as shown in the per−residue EDA of F252 to each ligand (Figure 4E).

#### 2.4.2. MD Simulations of MMP-13 Complexed with Ligands Showed the Importance of the π−π Stacking and π−CH(Cβ) Interactions with H222 and Y244, respectively

C103, C104, and C105 possess fused heterocyclic rings, such as pyrrole and pyrazole, and they can form π−π stacking interactions with F252 in a similar way to C101 with the fused thiophene ring. However, their inhibitory effects on MMP−13 were substantially dropped to >5000–274 nM IC_50’_s, implying that another factor may exist to affect the inhibition of ligands against MMP−13. Interestingly, C103 had two NH units in the 2−amino-pyrimidin−4−one scaffold (N4 and N6 in Figure 4A(c)), making the additional H−bond interactions with the amide backbone (C=O) of T245 (Table 2). However, its inhibition against MMP−13 notably disappeared (IC_50_ = >5000 nM). The MD simulation showed the formation of the H−bond interaction between N5 of C103 or C104 (Figure 4A(c,d)) with the amide backbone of G237 or A238, and such interaction led to the further shift of the ligand to the S1′−site (Figure 4F). Consequently, this movement of the ligand displaced the face−to−face stacking interaction of the 2-fluorophenyl ring initially sandwiched between H222 and Y244 (blue circle in Figure 4F). Particularly, both N4 and N6 units of C103 (Figure 4A(c)) shared the H−bond interaction with the amide backbone (C=O) of T245 (Table 2), resulting in the sizable displacement of its 2−fluorophenyl ring from H222 and Y244. Since the inhibition potencies of C103 and C104 were remarkably lowered, the face−to−face stacking and hydrophobic−π interactions of the ligand with H222 and Y244 are critical for the inhibition against MMP−13, rather than H−bond interactions with the specificity loop in the S1′−site. Similarly, C107, possessing N4 and N5 units in its scaffold (Figure 4A(f)), formed the H-bond interactions with the amide backbone of T245 (Table 2), reducing the stacking interaction with H222 and Y244 and inhibitory effect to MMP−13.

## 3. Discussion

The S1′−sites of MMPs are the most diverse among the six binding pockets in MMPs. Since the S1′−site is encompassed by the specificity loop, it has been known to determine the substrate specificity of MMPs [41]. In addition, due to the distinct nature of the pocket and the loop, the S1′−site has been considered a desired pocket to address the selectivity of ligands inhibiting each MMP. However, the systematic analysis of the specificity loop using a set of S1′−site binders has rarely been conducted, possibly due to the lack of such agents with their biological activities. Herein we analyzed the specificity loop of MMP−13 with ligands possessing various scaffolds occupying the S1′−site by MD simulations to identify important amino acids and their characteristics for ligand binding to the S1′−site.

### 3.1. The T247 Residue Contributes to the Formation of the Hydrophobic S1′−Site in Solution, but Its Conformation Can Be Varied, Depending on a Ligand in the S1′−Site

**Figure 5:**
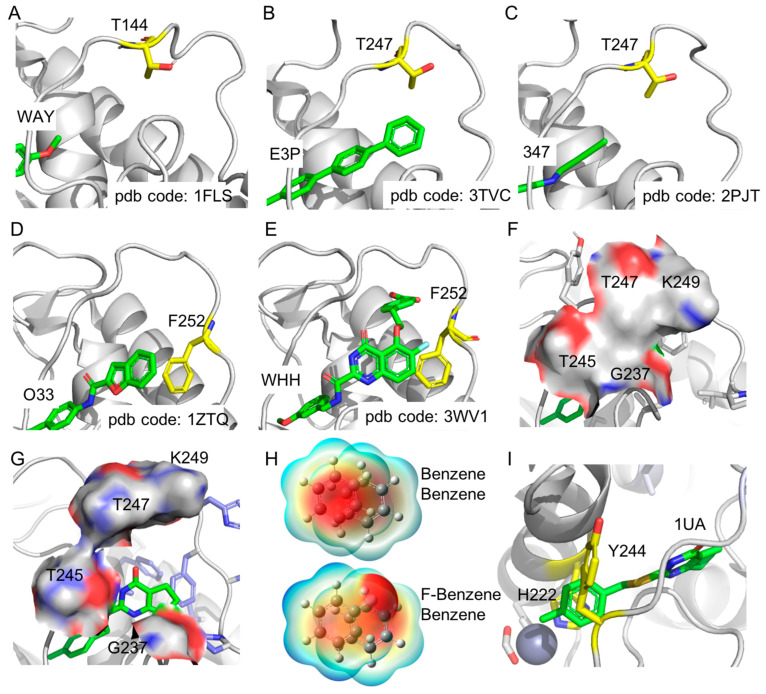
The specificity loop and S1′−site of MMP−13 complexed with ligands: (**A**–**C**) The NMR structure of MMP−13 (pdb code: 1FM1) and the X-ray co-crystal structures of MMP−13 (pdb codes: 3TVC and 2PJT), respectively, which shows the flexible conformation of T144/T247 (yellow). The part of each ligand in the S1′-site is shown in green, and each ligand code is labeled; (**D**,**E**) The edge−to−face and offset π−π stacking interactions between F252 and ligands in the S1′−site of MMP−13, respectively; (**F**) The S1′−site closed by the hydrophobic surface made by G237, T245, T247, and K249 in the specificity loop; (**G**) The open state of S1′−site; (**H**) The electrostatic potential surface of the offset π–π stacking interaction of benzene and fluorobenzene; (**I**) The sandwiched π−π and π−CH (Cβ) interactions by the ligand, H222, and Y244, which could be critical for the ligand binding to MMP−13.

The MD simulations of apo−MMP−13 show that the N−terminal portion of the specificity loop is well conserved, while its C−terminal portion is fairly flexible due to the frequent conformational change of the F252 residue during the simulations. However, once the F252 residue is located in a place to maximize van der Waals interactions in the S1′−site, the loop becomes conserved without significant conformational changes. Interestingly, the methyl unit of T247 is mostly oriented to the S1′−site and participates in the formation of the hydrophobic surface area in the S1′−site during the MD simulations. The analysis of currently available MMP−13 structures in the PDB database shows that one NMR structure (PDB code: 1FLS) has a similar conformation of T247 in the MD simulation (T144 in the 1FLS structure, Figure 5A) [42]. In addition, another NMR structure (PDB code: 1FM1) contains 30 conformations of MMP−13, and 21 out of 30 conformers have the methyl unit of T247 (T144 in the 1FM1) oriented to the S1′−site to maximize the hydrophobic contacts [42]: the other NMR structure (PDB code: 1EUB) is not included in this analysis due to the different conformation of its specificity loop [35]. However, only two out of 52 X-ray co−crystal structures of MMP−13 have the methyl unit of T247 pointed to the S1′−site (PDB codes: 3TVC and 2PJT) [43,44], while others have various conformations. The MMP−13 structure, coded as 3TVC, includes a L−glutamate motif inhibitor (ligand code: E3P), and its terphenyl moiety is reached out to the S1′−site to form hydrophobic interactions in the site (Figure 5B). Thus, the methyl unit of T247 could contribute to the formation of hydrophobic surface area in the S1′−site to enhance the binding affinity of the inhibitor. In addition, the MMP−13 structure, coded as 2PJT, includes four chains, and one of them shows that the hydroxyl group of T247 rather than the methyl unit is oriented to the S1′−site, implying its flexibility, since the methylacetylene unit of the inhibitor (ligand code: 347) could not reach the S1′−site (Figure 5C). Therefore, the T247 residue could participate in the formation of the hydrophobic surface area in the S1′−site in the solution state. However, the conformation of the T247 residue can be easily switched depending on a ligand or a moiety of a ligand in the S1′−site.

### 3.2. The F252 Residue Is Flexible and Contributes to π−Involved Interactions with the Ligand in the S1′−Site of MMP−13

All of the current MMP−13 X-ray co-crystal structures show that the phenyl ring of F252 forms edge−to−face stacking interaction with the F217 residue. However, the MD simulations of apo−MMP−13 structure show no meaningful π interactions mediated by the F252 residue in the S1′ site, such as π−π, CH−π, or edge−to−π interactions [45,46], and the F252 residue frequently changes its conformations to find the optimum hydrophobic contacts in the S1′−site. Therefore, the S1′−site is accessible by hydrophobic ligands, and the F252 residue can provide π−involved interactions with the upcoming ligands. For example, benzofuran or terphenyl of a ligand (ligand code: O33/518 or E3P, respectively) complexed with MMP−13 (PDB code: 1ZTQ/3I7I or 3TVC) forms the edge−to−face stacking with the F252 residue (Figure 5D) [43,47,48]. In addition, the offset π−π interactions between the ligand’s aromatic rings and the F252 residue are present in MMP−13 X-ray co−crystal structures (PDB code: 3WV1, 3WV2, 3WV3, 4L19, 5UWL, 5UWN, Figure 5E) [8,38,49,50]. Since the phenylalanine (F252) is highly conserved in other MMPs, it is uncertain to address MMP−13 specificity by targeting F252−mediated interactions. However, it would be a valuable approach to compare the dynamics of the phenyl ring of the residue in other MMPs, since distinct amino acids are connected or neighbored to the phenylalanine in the specificity loops of MMPs [10]. Our MD simulations of MMP−13 − ligands prove the importance of F252−mediated π interactions for the ligand binding affinity, and ligands that could not have stacking interactions with F252 have reduced inhibition against MMP−13 [13]. A ligand (1UA) is a highly selective inhibitor of MMP−13 vs. other MMPs, and it has CHπ interaction with the F252 residue of MMP−13 [38]. Therefore, F252 may have a distinct conformation or provide different contributions to the ligand binding in the S1′−site of each MMP, although it is highly conserved among MMPs.

### 3.3. The T247 Residue Has Dual Roles for the Ligand Binding to the S1′−Site by Forming the H−Bond Interaction and the Hydrophobic Surface Covering the S1′−Site

MD simulations show that T247 plays roles in the ligand binding to the S1′−site as a H−bond donor by the hydroxyl group and a donor for the van der Waals surface by the methyl group. Due to the dual roles of T247, the specificity loop of MMP−13 can have a closed conformation, which could block the access of water molecules to the S1′−site, leading to the stabilized ligand (1UA) in the S1′−site of MMP−13 (Figure 5F). The methyl unit of T247 participates in the formation of the van der Waals surface in the S1′−site in the MD simulations of apo−MMP−13. However, the ligand binding to the S1′−site can cause its conformational change to maximize the ligand stability in the pocket. Although the hydrophobic surface composed of two threonine residues (T245 and T247) is small, it is sufficient to shield the ligand bound to the S1′−site due to the presence of a loop and the methylene unit of K249 (Figure 5F). This surface can be compared to that in the open conformation of the loop obtained from the MD simulations (Figure 5G). Thus, the specificity loop of MMP-13 could not only provide a ligand with components for the H-bond interactions but also form an additional van der Waals surface to make the ligand stable in the site by hampering the access of water molecules to the S1′−site.

### 3.4. The Offset π−π Stacking and π−CH(Cβ) Interactions Are Important for the Ligand Binding to the S1′−Site of MMP−13

The offset and edge−to−face π−π stacking interactions are more common in the protein folding and protein−ligand interactions than face−to−face interactions, and those interactions have been applied to the design of ligands with higher binding affinities [51]. A ligand (C100) with the 6−fluoroquinazolin−4−one scaffold has a more favored interaction with the F252 residue than a ligand (C99) without fluorine at the 6−position. The QM calculations also show a better binding affinity between fluorobenzene and benzene than benzene and benzene in the offset conformation. This result corresponds to the experimental assay data with 4−fold higher inhibition potency of C100 against MMP−13 than C99. Since there is little overlap between two rings, such as the fused phenyl ring of quinazolin−4−one and the phenyl ring of F252, the negative electrostatic potential on the fluorine atom could produce a more favorable interaction with the positive potential around the periphery of the F252 residue (Figure 5H).

The 4−methylphenyl ring of 1UA also forms offset π−π stacking interaction with imidazole of H222, of which δ−N is parallel to the center of the phenyl ring of 1UA in the X-ray co−crystal structure of MMP−13 (Figure 5I). Although the face−to−face stacking conformation of these aromatic rings is frequently detected in the MD simulations, the MMP−13 − 1UA complex structures with the lowest potential energy in all simulations have off−set stacking conformation only. Therefore, the negative electrostatic potential of imidazole nitrogen can play a role as an aromatic donor, and the phenyl ring of 1UA does so as an aromatic acceptor in the complex form [40]. This donor/acceptor relationship can be confirmed by the 4−times enhanced inhibition potency of C2 (with fluorine on the phenyl ring) against MMP−13, compared to C1 without any fluorine on the ring.

The phenyl ring of 1UA is involved in the π and CH (Cβ) interaction with Y244 (Figure 5I) [52,53]. The CH (aliphatic) and π interaction is one of the hydrophobic contacts in protein−ligand complexes. The systematic analysis of protein−ligand interactions in the PDB database shows that the hydrophobic contacts between the aliphatic CH of hydrophobic residues and the aromatic ring of a ligand are most frequently observed in protein−ligand complexes [51]. However, its interactions between the aliphatic CH (Cβ) of tyrosine/phenylalanine/tryptophan and the aromatic ring of ligands have been rarely analyzed. In the X-ray co−crystal structure of MMP−13 and 1UA, the phenyl ring of 1UA is sandwiched between imidazole of H222 and CH (Cβ) of Y244 via offset π−π stacking and π−hydrophobic interactions, respectively (Figure 5I). In the MD simulations, C103 binds to MMP−13 by shifting to the S1′−site due to the H−bond interactions by two NH units of the ligand, and it breaks offset stacking and π−CH (Cβ) interactions with H222 and Y244. Considering the significantly decreased inhibition potency of C103 against MMP−13, the sandwiched π−π stacking and π−CH (Cβ) interactions with H222 and Y244 could be essential for the ligand binding to MMP−13. A recent X-ray co−crystal structure of MMP−13 in complex with a fragment, 4−(1,2,3-thiadiazole−4−yl)pyridine (ligand code: 9DY) shows that the pyridine ring of the fragment forms the offset π−π stacking interaction with H222 in the tunnel area (PDB code: 7JU8, Figure S4) [54]. Due to the presence of nitrogen on the pyridine of the fragment, its offset pattern is different from that of the phenyl ring of 1UA, and the pyridine ring is more shifted to the Zn−binding site, resulting in the offset π−CH (Cβ) interactions with Y244. Since there are many plausible binding pockets in the target enzyme that such a small fragment can occupy, the inserted aromatic ring between H222 and Y244 in the tunnel area is the vital moiety of a ligand for its binding to MMP-13.

## 4. Materials and Methods

### 4.1. Protein Preparation

The X-ray co−crystal structure of MMP−13 − 1UA was retrieved from the Protein Data Bank (PDB code: 4L19), and chain A, all water molecules except two coordinated to a Ca ion, and the N−terminal amino acids (Y104−R109) were deleted to clean up the structure. The structure was further refined with Protein Preparation Wizard implemented in Maestro 12 (Schrödinger Inc., New York, NY, USA). The protein structure was imported into the workspace and preprocessed to assign bond orders, add hydrogen atoms, create zero-order bonds to metals, and create disulfide bonds. In addition, missing atoms in residues and missing loops were added using Prime to generate a complete protein structure. The N− and C−termini were capped with ACE and NME, and cofactors such as formic acid (FMT), except the one coordinated to a Zn ion and glycerol (GOL). were deleted. The protein structure was further refined via automated H−bond assignment and restrained minimization with an OPLS 2005 force field by converging heavy atoms to 0.3 Å RMSD. Based on the protein preparation of the 4L19 structure in Maestro 12, H131, H157, H200, and H251 were protonated at the delta position, and H172, H187, H222, H226, andH232 were protonated at the epsilon position. No disulfide bond or seleno−methionine residue was identified in the structure. The structure was cleaned according to the protonation states and prepared for building protein systems by treating metals and their binding sites.

### 4.2. Parameterization of Metal Sites

The parameters of metal sites were generated by following the procedures in the Amber tutorials using the python−based Metal Center Parameter Builder (MCPB.py) [37]. First, the PDB, mol2, and parameter modification (frcmod) files for the non−standard residues such as Zn, CA, water, and FMT were generated by using antechamber, tleap, and parmchk2 commands and a metalpdb2mol2.py script. All pdb files were combined into a single pdb file in a sequence of a protein, Zn and Ca ions, FMT, and water molecules, and the pdb4amber command was used to renumber the pdb file. The MCPB.py (step 1) was used to generate input files of small and large models for Gaussian09 calculations and fingerprint modeling files. For the small model, the optimization of all atoms was initially conducted. However, since there were four ions, such as two Zn and two Ca ions, in the MMP−13, the whole system included 229 atoms in the small model, and the hydrophobic moieties of the simplified amino acids were gathered together during the optimization, resulting in an irreverent structure. Thus, the partial optimization of each ion and its coordinated residues was conducted using the B3LYP/6−31G* level of theory and the basis sets. For example, Zn 1 ion and atoms in the Zn 1−coordinated residues were optimized, while other atoms were frozen. The partially optimized geometry was extracted, and Zn 2 ion and atoms in the Zn 2−coordinated residues were optimized, while other atoms were frozen. This partial optimization procedure was conducted for four metal ions. For the large model, the Merz−Kollman RESP charge calculation was performed [55]. The Seminario method was applied by using the MCPB.py (step 2) to generate the bond and angle force field parameters for the metal site residues. In the next MCPB.py procedure (step 3), the ChgModB method was used to conduct the RESP charge fitting to generate mol2 files, of which charges were refitted by the MK RESP charge fitting algorithm. In the last procedure (step 4), a tleap input file was generated. In the generated tleap file, two lines were deleted, “bond mol.169.CA mol.172.O” and “bond mol.169.CA mol.173.O”, for two bonds between the Ca 1 ion and oxygen atoms of two water molecules. These bonds were treated in a nonbonded way by using the restrained nonbonded model. The harmonic restraints for the restrained nonbonded model of Ca 1 ion and two water molecules were generated based on the metal ion related bond information by using ParmEd (printBonds: CA1 command). Lastly, a line containing TER above the lines for the NME residue in the generated pdb structure (4L19_mcpbpy.pdb) was deleted.

### 4.3. System Buildup and MD Simulations Using Amber20

The structure generated from the above metal site parameterization was solvated with the Amber ff19SB force field and TIP3P explicit water model in the periodic box using a buffer distance of 12.0 Å containing 150 mM NaCl in water molecules. After the topology and coordinate files were generated by tleap, the structure, particularly each metal site, was visually inspected in VMD 1.9.4a43 [56], and ParmEd was also used to examine the metal site parameters. The minimization of the system was conducted in three consecutive steps: 1000 steps of steepest descent and 1000 steps of conjugate gradient minimization of water molecules only, the same minimization steps for solute only, and 2500 and 2500 steps for the whole system, respectively. After the minimization, the system was heated from 0 K to 298.15 K for 300 ps and equilibrated at the same temperature for 100 ps under constant volume, with a timestep of 1 fs, and with SHAKE algorithm employed to restrain the calculation of forces of bonds containing hydrogen atoms. The system was further equilibrated at 298.15 K for 25 ps under constant pressure with a Berendsen barostat and Langevin thermostat: 5 times for the 5 ps equilibration run with the latest velocity and coordinates saved from the previous run. After the equilibration, the system was simulated in the production step at 298.15 K for 100 ns under the NPT condition: 10 simulations, where each simulation is 10 ns long. For each new simulation, the final velocities and coordinates from the previous simulation were used as the starting velocities and coordinates of the new simulation (continuous trajectory).

### 4.4. Trajectory Analysis with CPPTRAJ and Python Scripts

The simulation data were analyzed with CPPTRAJ by reading and combining multiple trajectory files obtained from the MD simulations [57,58]. The protein structures in the trajectories were re−oriented by using the autoimage command, and water molecules, Na, Cl ions were striped. RMS fit of all residues was performed, and a new NetCDF trajectory was saved for further analysis. For the rmsd analysis, the rms and atomicfluct commands were used, and the amide backbones, Cα, and Cβ without hydrogen of M116−Y266 were selected for the analyses of rmsd and fluctuation. An in−house python script for the rmsd data was used to generate Figure 1B. For the histogram analysis of dihedral angle distributions, the multidihedral and multihist commands were used to generate the data of phi, psi and chi dihedral angles and their histograms. The python script was used to generate Figure 1D,G. The H−bond interactions between the protein and the ligand were analyzed using the hbond command, and a python script and gnuplot were used to generate figures (Figure 2C and Figure 3B).

### 4.5. Relative Binding Free Energy Calculation by TI Simulations

The relative binding free energy of C1 to C2A or C2B transformation (ΔΔG_(C1 to C2A)_ or ΔΔG_(C1 to C2B)_) was calculated as the difference between the free energy of changing C1 to C2A or C2B in MMP−13 and in solution. Hence, TI simulations for the process of transformation in the MMP−13 and in solution were performed. The 3−step protocol was applied for both processes by removing charge on hydrogen (H28) of C1, changing van der Waals and bonded terms (H28 to F1), and recharging fluorine (F1) of C2. The restrained electrostatic potential (RESP) charge for C1, C2A, and C2B were calculated at the B3LYP/6−31G* level of theory and basis sets using the Gaussian 09. The antechamber and the general Amber force field 2 (GAFF2) were used for the parameterization of ligands, and the parmchk2 was used for the missing parameters of ligands. The equilibrated structure of MMP−13 − C1 obtained from the 100 ns MD simulation was used as a starting structure for the simulations in 10 windows (λ, 0.0–1.0 with 0.1 intervals). For each λ, the system was minimized for 1000 steps using the steepest descent method and heated from 0 to 298.15 K for 50 ps (after 2 ps simulation at 10 K) with a timestep of 1 fs by using the Langevin thermostat with a collision frequency of 1.0 ps^−1^. Afterward, the system was equilibrated to 35 ps (5 times of 7 ps) of NPT. Lastly, 4 ns of NPT simulation was performed (2 times 2 ns), and dV/dλ was collected for every 2 ps of the last 2 ns for each simulation. The TI gradients were integrated by a linear extrapolation to compute ΔG for ligands and complex, and ΔΔGs were calculated by subtracting ΔG_ligands_ from ΔG_complex_.

### 4.6. MM/GBSA Calculations and Analysis

All of the MM/GBSA energies were calculated based on snapshots (frames) extracted from MD trajectories obtained from the simulations (Section 4.3). Around 1000 snapshots in the last 10 ns simulation of each ligand were used for calculations, and the saltcon (the concentration of mobile counterions in solution) was set to 0.150. The ΔG_MM/GBSA_ for each ligand was obtained from the result data file for the analysis. Amino acids within 5 Å of a ligand (C1) were selected for the per−residue energy decomposition analysis, and energies were decomposed on a pairwise per−residue basis (idecomp = 4). An in−house python script was used to generate figures (Figure 2G, Figure 3A and Figure 4E) for the visual analysis of per−residue energies to each ligand. Vibrational entropies were approximated by the normal mode calculations, and 20 snapshots in the last 10 ns simulation were used for the estimation of ΔG_binding_ with the entropy term.

### 4.7. Quantum Mechanical Calculations

Quantum Mechanical (QM) calculations were carried out using the Gaussian 16 software version ES64L−G16RevC.01[59] to obtain the optimized geometry and binding free energy of π−π stacking interactions between aromatic rings on the ligands and the aromatic rings of phenylalanine and histidine. To simply the system, benzene/benzene and benzene/fluorobenzene complex, which mimic the interaction between phenylalanine and quinazolin−4−one (or 6−fluoroquinazolin−4−one), were simulated in the QM calculation. The initial coordinates of the aromatic rings were taken from the X-ray crystal structure of MMP−13 complexed with the 1UA inhibitor. A geometry optimization was then performed using the MP2/6−31+G(d,p) method and basis sets. Several Density Functional Theory (DFT) methods were initially used to optimize the geometry of the complex system, but these methods yielded varying structures, all of which were not synonymous with π−π stacking interactions. After the geometry optimization, a vibrational frequency calculation was performed using the same method and basis sets to confirm the stationary point and calculate the free energy of the system. The free energies for each aromatic ring and their complex were used to calculate the ΔG values by using the following equation.

ΔG = G_complex_ − ΣG_component_

where G_complex_ is the free energy for a multi ring system, and G_component_ is the free energy for one component of that system. The resulting ΔG was then converted from Hartrees to kcal/mol.

## 5. Conclusions

From the MD simulations of apo−MMP−13 and its complex state with various ligands, we can identify the dual role of T247 for the ligand binding to the S1′−site. Particularly, the hydrophobic surface made by T247, T245, and K249 generates the pocket−like S1′−site to stabilize a ligand in the site. In addition, the F252 residue, although it frequently rotates in the apo − state, could provide the offset π−π or edge−to−π stacking interactions to the ligand in the S1′−site. Interestingly, the aromatic ring of a ligand could be inserted between H222 and Y244 through the offset π−π and π−CH(Cβ) interactions in the tunnel area. We anticipate that these findings will provide insights for the discovery of S1′−site binders of MMP−13 and other MMPs, leading to the development of selective inhibitors of MMPs via computer−aided drug design efforts. Further systematic analysis of the offset π−π and π−CH(Cβ) interactions with ligands having various aromatic rings connected to cyclopentapyrimidin−4−one could be a valuable approach to understanding such interactions in the biomolecular systems. In addition, ligands having quinazolin−4−one derivatives could be an interesting set of compounds to conduct the systematic analysis of the offset π−π stacking interaction in the S1′−site of MMPs. The results of these studies will be reported in due course.

## Figures and Tables

**Table 1:** Two distinct binding poses of 1UA and C1 to MMP−13 and their estimated binding free energies.

	Binding Pose 1 (Figure 2E)		Binding Pose 2 (Figure 2F)	
	ΔG_binding_^a^	ΔG_MM/GBSA_^b^	ΔG_binding_	ΔG_MM/GBSA_
1UA	−13.3 (2.9) ^c^	−35.7 (2.0) ^c^	−20.6 (4.2) ^c^	−42.2 (2.5) ^c^
C1	−16.1 (5.6) ^c^	−44.9 (2.7) ^c^	−22.1 (5.7) ^c^	−50.6 (4.3) ^c^
interactions	van der Waals by CH_3_ of T247		H−bond by OH of T247	

^a^ The binding free energy with the normal mode entropy approximation. ^b^ The binding free energy without the entropy approximation. ^c^ Units are kcal/mol; standard deviations are in parenthesis.

**Table 2:** H−bond analysis of ligands C99−C110.

Ligands	Scaffold ^a^	Subst. (X) ^a^	IC_50_ (nM) ^b^	Acceptor ^c^	DonorH ^d^	Fraction
C99	a	H	9.4	T245_O	LIG_N4H	0.9135
				LIG_O	T247_OH	0.2518
C100	a	F	2.5	T245_O	LIG_N4H	0.9337
				LIG_O	T247_OH	0.6417
C101	b	H	8.4	T245_O	LIG_N4H	0.9315
				LIG_O	T247_OH	0.4352
C102	b	CH_3_	13	T245_O	LIG_N4H	0.9112
				LIG_O	T247_OH	0.4918
C103	c	−	>5000	T245_O	LIG_N4H	0.6115
				T245_O	LIG_N6H	0.4205
				LIG_O	T247_OH	0.4390
				G237_O	LIG_N5H	0.4810
C104	d	C	2000	T245_O	LIG_N4H	0.8538
				LIG_O	T247_OH	0.6893
				A238_O	LIG_N5H	0.4672
C105	d	N	274	T245_O	LIG_N4H	0.8290
				LIG_O	T247_OH	0.5870
				A238_O	LIG_N5H	0.6752
C106	e	X=H Y=H	153	T245_O	LIG_N4H	0.9108
				LIG_O	T247_OH	0.7200
C107	f	−	2600	T245_O	LIG_N4H	0.0458
				LIG_O	T247_OH	0.0508
				T245_O	LIG_N5H	0.7390
C108	e	X=CH_3_ Y=H	1300	T245_O	LIG_N4H	0.9297
				LIG_O	T247_OH	0.4493
C109	e	X=H Y=CH_3_	88	T245_O	LIG_N4H	0.9367
				LIG_O	T247_OH	0.5833
C110	e	X=H Y=CF_3_	1200	T245_O	LIG_N4H	0.8892
				LIG_O	T247_OH	0.1833

^a^ Structures are in Figure 4A. ^b^ IC_50_ values were obtained from a reference [13]. ^c^ LIG_O and T245_O/G237_O/A238_O stand for carbonyl oxygen in the pytimidin−4−one scaffold of ligands and the amide backbone of each amino acid, respectively. ^d^ LIG_N4H, LIG_N5H, and LIG_N6H stand for hydrogen of each ligand labelled in Figure 4A, and T247_OH for hydrogen (OH) of the T247 residue.

## Data Availability

The raw data are available on request from the authors.

## Supplementary Materials

The following supporting information can be downloaded at: https://www.mdpi.com/article/10.3390/ijms241310577/s1.

## References

[1] de Almeida L.G.N., Thode H., Eslambolchi Y., Chopra S., Young D., Gill S., Devel L., Dufour A. (2022). Matrix Metalloproteinases: From Molecular Mechanisms to Physiology, Pathophysiology, and Pharmacology. Pharmacol. Rev..

[2] Winkler J., Abisoye-Ogunniyan A., Metcalf K.J., Werb Z. (2020). Concepts of extracellular matrix remodelling in tumour progression and metastasis. Nat. Commun..

[3] Diaz-Canestro C., Puspitasari Y.M., Liberale L., Guzik T.J., Flammer A.J., Bonetti N.R., Wust P., Costantino S., Paneni F., Akhmedov A. (2022). MMP-2 knockdown blunts age-dependent carotid stiffness by decreasing elastin degradation and augmenting eNOS activation. Cardiovasc. Res..

[4] Visse R., Nagase H. (2003). Matrix metalloproteinases and tissue inhibitors of metalloproteinases: Structure, function, and biochemistry. Circ. Res..

[5] Fields G.B. (2019). The Rebirth of Matrix Metalloproteinase Inhibitors: Moving Beyond the Dogma. Cells.

[6] Li K., Tay F.R., Yiu C.K.Y. (2020). The past, present and future perspectives of matrix metalloproteinase inhibitors. Pharmacol. Ther..

[7] Lenci E., Cosottini L., Trabocchi A. (2021). Novel matrix metalloproteinase inhibitors: An updated patent review (2014–2020). Expert Opin. Ther. Pat..

[8] Choi J.Y., Fuerst R., Knapinska A.M., Taylor A.B., Smith L., Cao X., Hart P.J., Fields G.B., Roush W.R. (2017). Structure-Based Design and Synthesis of Potent and Selective Matrix Metalloproteinase 13 Inhibitors. J. Med. Chem..

[9] Roth J., Minond D., Darout E., Liu Q., Lauer J., Hodder P., Fields G.B., Roush W.R. (2011). Identification of novel, exosite-binding matrix metalloproteinase-13 inhibitor scaffolds. Bioorg. Med. Chem. Lett..

[10] Engel C.K., Pirard B., Schimanski S., Kirsch R., Habermann J., Klingler O., Schlotte V., Weithmann K.U., Wendt K.U. (2005). Structural basis for the highly selective inhibition of MMP-13. Chem. Biol..

[11] Fuerst R., Choi J.Y., Knapinska A.M., Cameron M.D., Ruiz C., Delmas A., Sundrud M.S., Fields G.B., Roush W.R. (2022). Development of a putative Zn(2+)-chelating but highly selective MMP-13 inhibitor. Bioorg. Med. Chem. Lett..

[12] Fabre B., Ramos A., de Pascual-Teresa B. (2014). Targeting matrix metalloproteinases: Exploring the dynamics of the s1′ pocket in the design of selective, small molecule inhibitors. J. Med. Chem..

[13] Fuerst R., Yong Choi J., Knapinska A.M., Smith L., Cameron M.D., Ruiz C., Fields G.B., Roush W.R. (2018). Development of matrix metalloproteinase-13 inhibitors—A structure-activity/structure-property relationship study. Bioorg. Med. Chem..

[14] Setoh M., Ishii N., Kono M., Miyanohana Y., Shiraishi E., Harasawa T., Ota H., Odani T., Kanzaki N., Aoyama K. (2014). Discovery of the first potent and orally available agonist of the orphan G-protein-coupled receptor 52. J. Med. Chem..

[15] Case D.A., Cheatham T.E., Darden T., Gohlke H., Luo R., Merz K.M., Onufriev A., Simmerling C., Wang B., Woods R.J. (2005). The Amber biomolecular simulation programs. J. Comput. Chem..

[16] Cournia Z., Allen B., Sherman W. (2017). Relative Binding Free Energy Calculations in Drug Discovery: Recent Advances and Practical Considerations. J. Chem. Inf. Model..

[17] Mobley D.L., Klimovich P.V. (2012). Perspective: Alchemical free energy calculations for drug discovery. J. Chem. Phys..

[18] King E., Aitchison E., Li H., Luo R. (2021). Recent Developments in Free Energy Calculations for Drug Discovery. Front. Mol. Biosci..

[19] Lee T.S., Allen B.K., Giese T.J., Guo Z., Li P., Lin C., McGee T.D., Pearlman D.A., Radak B.K., Tao Y. (2020). Alchemical Binding Free Energy Calculations in AMBER20: Advances and Best Practices for Drug Discovery. J. Chem. Inf. Model..

[20] Carrascal N., Rizzo R.C. (2009). Calculation of binding free energies for non-zinc chelating pyrimidine dicarboxamide inhibitors with MMP-13. Bioorg. Med. Chem. Lett..

[21] Huang S., Feng K., Ren Y. (2019). Molecular modelling studies of quinazolinone derivatives as MMP-13 inhibitors by QSAR, molecular docking and molecular dynamics simulations techniques. Medchemcomm.

[22] Mathpal S., Sharma P., Joshi T., Pande V., Mahmud S., Jeong M.K., Obaidullah A.J., Chandra S., Kim B. (2022). Identification of Zinc-Binding Inhibitors of Matrix Metalloproteinase-9 to Prevent Cancer Through Deep Learning and Molecular Dynamics Simulation Approach. Front. Mol. Biosci..

[23] Khandelwal A., Lukacova V., Comez D., Kroll D.M., Raha S., Balaz S. (2005). A combination of docking, QM/MM methods, and MD simulation for binding affinity estimation of metalloprotein ligands. J. Med. Chem..

[24] Varghese A., Chaturvedi S.S., DiCastri B., Mehler E., Fields G.B., Karabencheva-Christova T.G. (2021). Effects of the Nature of the Metal Ion, Protein and Substrate on the Catalytic Center in Matrix Metalloproteinase-1: Insights from Multilevel MD, QM/MM and QM Studies. Chemphyschem.

[25] Waheed S.O., Varghese A., DiCastri I., Kaski B., LaRouche C., Fields G.B., Karabencheva-Christova T.G. (2023). Mechanism of the Early Catalytic Events in the Collagenolysis by Matrix Metalloproteinase-1. Chemphyschem.

[26] Nash A., Birch H.L., de Leeuw N.H. (2017). Mapping intermolecular interactions and active site conformations: From human MMP-1 crystal structure to molecular dynamics free energy calculations. J. Biomol. Struct. Dyn..

[27] Karabencheva-Christova T.G., Christov C.Z., Fields G.B. (2018). Conformational Dynamics of Matrix Metalloproteinase-1.Triple-Helical Peptide Complexes. J. Phys. Chem. B.

[28] Singh W., Fields G.B., Christov C.Z., Karabencheva-Christova T.G. (2016). Importance of the Linker Region in Matrix Metalloproteinase-1 Domain Interactions. RSC Adv..

[29] Singh W., Fields G.B., Christov C.Z., Karabencheva-Christova T.G. (2016). Effects of Mutations on Structure-Function Relationships of Matrix Metalloproteinase-1. Int. J. Mol. Sci..

[30] Varghese A., Chaturvedi S.S., Fields G.B., Karabencheva-Christova T.G. (2021). A synergy between the catalytic and structural Zn(II) ions and the enzyme and substrate dynamics underlies the structure-function relationships of matrix metalloproteinase collagenolysis. J. Biol. Inorg. Chem..

[31] Chen B., Kang Z., Zheng E., Liu Y., Gauld J.W., Wang Q. (2021). Hydrolysis Mechanism of the Linkers by Matrix Metalloproteinase-9 Using QM/MM Calculations. J. Chem. Inf. Model..

[32] Vasilevskaya T., Khrenova M.G., Nemukhin A.V., Thiel W. (2015). Mechanism of proteolysis in matrix metalloproteinase-2 revealed by QM/MM modeling. J. Comput. Chem..

[33] Tallant C., Marrero A., Gomis-Ruth F.X. (2010). Matrix metalloproteinases: Fold and function of their catalytic domains. Biochim. Biophys Acta.

[34] Bode W., Gomis-Ruth F.X., Stockler W. (1993). Astacins, serralysins, snake venom and matrix metalloproteinases exhibit identical zinc-binding environments (HEXXHXXGXXH and Met-turn) and topologies and should be grouped into a common family, the ‘metzincins’. FEBS Lett..

[35] Zhang X., Gonnella N.C., Koehn J., Pathak N., Ganu V., Melton R., Parker D., Hu S.I., Nam K.Y. (2000). Solution structure of the catalytic domain of human collagenase-3 (MMP-13) complexed to a potent non-peptidic sulfonamide inhibitor: Binding comparison with stromelysin-1 and collagenase-1. J. Mol. Biol..

[36] Li P., Merz K.M. (2021). Parameterization of a Dioxygen Binding Metal Site Using the MCPB.py Program. Methods Mol. Biol..

[37] Li P., Merz K.M. (2016). MCPB.py: A Python Based Metal Center Parameter Builder. J. Chem. Inf. Model..

[38] Spicer T.P., Jiang J., Taylor A.B., Choi J.Y., Hart P.J., Roush W.R., Fields G.B., Hodder P.S., Minond D. (2014). Characterization of selective exosite-binding inhibitors of matrix metalloproteinase 13 that prevent articular cartilage degradation in vitro. J. Med. Chem..

[39] Genheden S., Ryde U. (2015). The MM/PBSA and MM/GBSA methods to estimate ligand-binding affinities. Expert Opin. Drug Discov..

[40] Pace C.J., Gao J. (2013). Exploring and exploiting polar-pi interactions with fluorinated aromatic amino acids. Acc. Chem. Res..

[41] Ratnikov B.I., Cieplak P., Gramatikoff K., Pierce J., Eroshkin A., Igarashi Y., Kazanov M., Sun Q., Godzik A., Osterman A. (2014). Basis for substrate recognition and distinction by matrix metalloproteinases. Proc. Natl. Acad. Sci. USA.

[42] Moy F.J., Chanda P.K., Chen J.M., Cosmi S., Edris W., Levin J.I., Powers R. (2000). High-resolution solution structure of the catalytic fragment of human collagenase-3 (MMP-13) complexed with a hydroxamic acid inhibitor. J. Mol. Biol..

[43] Devel L., Beau F., Amoura M., Vera L., Cassar-Lajeunesse E., Garcia S., Czarny B., Stura E.A., Dive V. (2012). Simple pseudo-dipeptides with a P2’ glutamate: A novel inhibitor family of matrix metalloproteases and other metzincins. J. Biol. Chem..

[44] Huang A., Joseph-McCarthy D., Lovering F., Sun L., Wang W., Xu W., Zhu Y., Cui J., Zhang Y., Levin J.I. (2007). Structure-based design of TACE selective inhibitors: Manipulations in the S1′–S3′ pocket. Bioorg. Med. Chem..

[45] Sherrill C.D. (2013). Energy component analysis of pi interactions. Acc. Chem. Res..

[46] Singh N.J., Min S.K., Kim D.Y., Kim K.S. (2009). Comprehensive Energy Analysis for Various Types of pi-Interaction. J. Chem. Theory Comput..

[47] Wu J., Rush T.S., Hotchandani R., Du X., Geck M., Collins E., Xu Z.B., Skotnicki J., Levin J.I., Lovering F.E. (2005). Identification of potent and selective MMP-13 inhibitors. Bioorg. Med. Chem. Lett..

[48] Heim-Riether A., Taylor S.J., Liang S., Gao D.A., Xiong Z., Michael August E., Collins B.K., Farmer B.T., Haverty K., Hill-Drzewi M. (2009). Improving potency and selectivity of a new class of non-Zn-chelating MMP-13 inhibitors. Bioorg. Med. Chem. Lett..

[49] Nara H., Sato K., Naito T., Mototani H., Oki H., Yamamoto Y., Kuno H., Santou T., Kanzaki N., Terauchi J. (2014). Thieno[2,3-d]pyrimidine-2-carboxamides bearing a carboxybenzene group at 5-position: Highly potent, selective, and orally available MMP-13 inhibitors interacting with the S1″ binding site. Bioorg. Med. Chem..

[50] Nara H., Sato K., Naito T., Mototani H., Oki H., Yamamoto Y., Kuno H., Santou T., Kanzaki N., Terauchi J. (2014). Discovery of novel, highly potent, and selective quinazoline-2-carboxamide-based matrix metalloproteinase (MMP)-13 inhibitors without a zinc binding group using a structure-based design approach. J. Med. Chem..

[51] Ferreira de Freitas R., Schapira M. (2017). A systematic analysis of atomic protein-ligand interactions in the PDB. Medchemcomm.

[52] Kumar M., Balaji P.V. (2014). C-H…pi interactions in proteins: Prevalence, pattern of occurrence, residue propensities, location, and contribution to protein stability. J. Mol. Model..

[53] Brandl M., Weiss M.S., Jabs A., Suhnel J., Hilgenfeld R.C.-H. (2001). Pi-interactions in proteins. J. Mol. Biol..

[54] Taylor S.J., Abeywardane A., Liang S., Xiong Z., Proudfoot J.R., Farmer B.S., Gao D.A., Heim-Riether A., Smith-Keenan L.L., Muegge I. (2021). Indole Inhibitors of MMP-13 for Arthritic Disorders. ACS Omega.

[55] Bayly C.I., Cianci C., Cornell W., Kollman P.A. (1993). A well-behaved electrostatic potential based method using charge restraints for deriving atomic charges: The RESP model. J. Phys. Chem..

[56] Humphrey W., Dalke A., Schulten K. (1996). VMD: Visual molecular dynamics. J. Mol. Graph..

[57] Roe D.R., Cheatham T.E. (2018). Parallelization of CPPTRAJ enables large scale analysis of molecular dynamics trajectory data. J. Comput. Chem..

[58] Roe D.R., Cheatham T.E. (2013). PTRAJ and CPPTRAJ: Software for Processing and Analysis of Molecular Dynamics Trajectory Data. J. Chem. Theory Comput..

[59] (2016). Gaussian 16.

