# Supplementary material for: Molecular Dynamics Simulations of Matrix Metalloproteinase 13 and the Analysis of the Specificity Loop and the S1′−Site
Source: Int J Mol Sci. 2023 Jun 24;24(13):10577. doi: 10.3390/ijms241310577 (PMC10342107; doi:10.3390/ijms241310577)
Supplement: Supplementary file 1 [file ijms-24-10577-s001.zip › ijms-2351303-supplementary.pdf]

Supporting Information

Molecular Dynamics Simulations of Matrix Metalloproteinase 13 and the Analysis of the Specificity Loop and the S1'-site

Jun Yong Choi [1,2,*], and Eugene Chung [1]

[1]Department of Chemistry and Biochemistry, Queens College, Flushing, NY 11367, USA
[2]Ph.D. Programs in Chemistry and Biochemistry, The Graduate Center of the City University of New York, New York, NY 10016, USA

*Correspondence: junyong.choi@qc.cuny.edu; Tel.: +1-718-997-3279

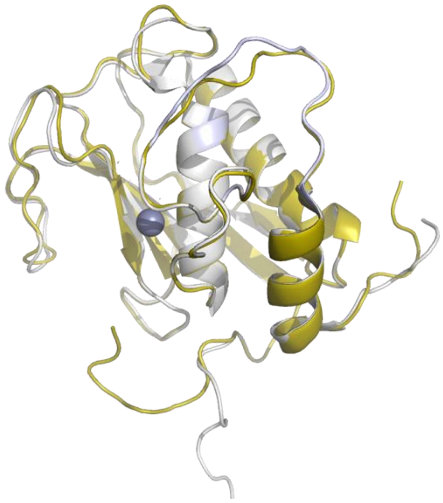

Figure S1. The structure alignment of apo-MMP-13 from the AlphaFold database (gold, identifier: AF-P45452-F1-model_v4) to the X-ray co-crystal structure of MMP-13 – 1UA complex (light blue, PDB code: 4L19)

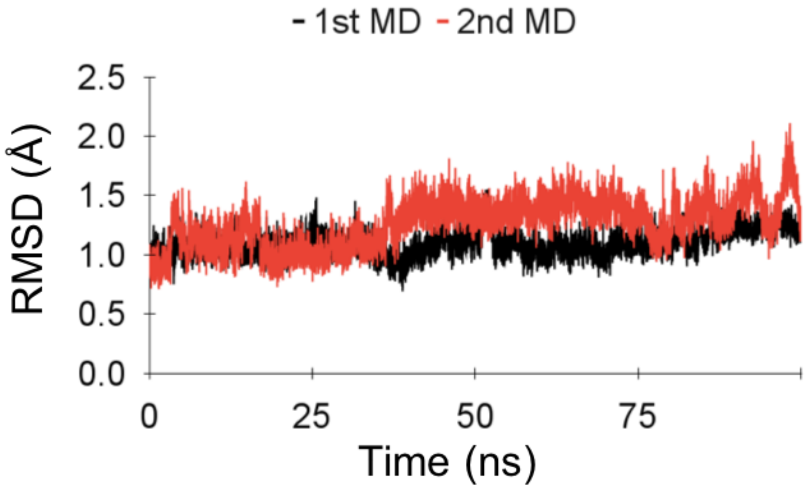

Figure S2. RMSD of two MD simulations of MMP-13 – 1UA complex.

A

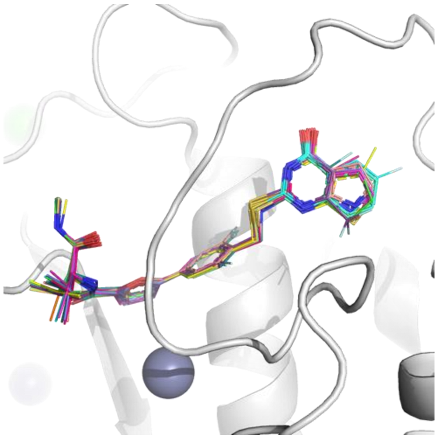

B

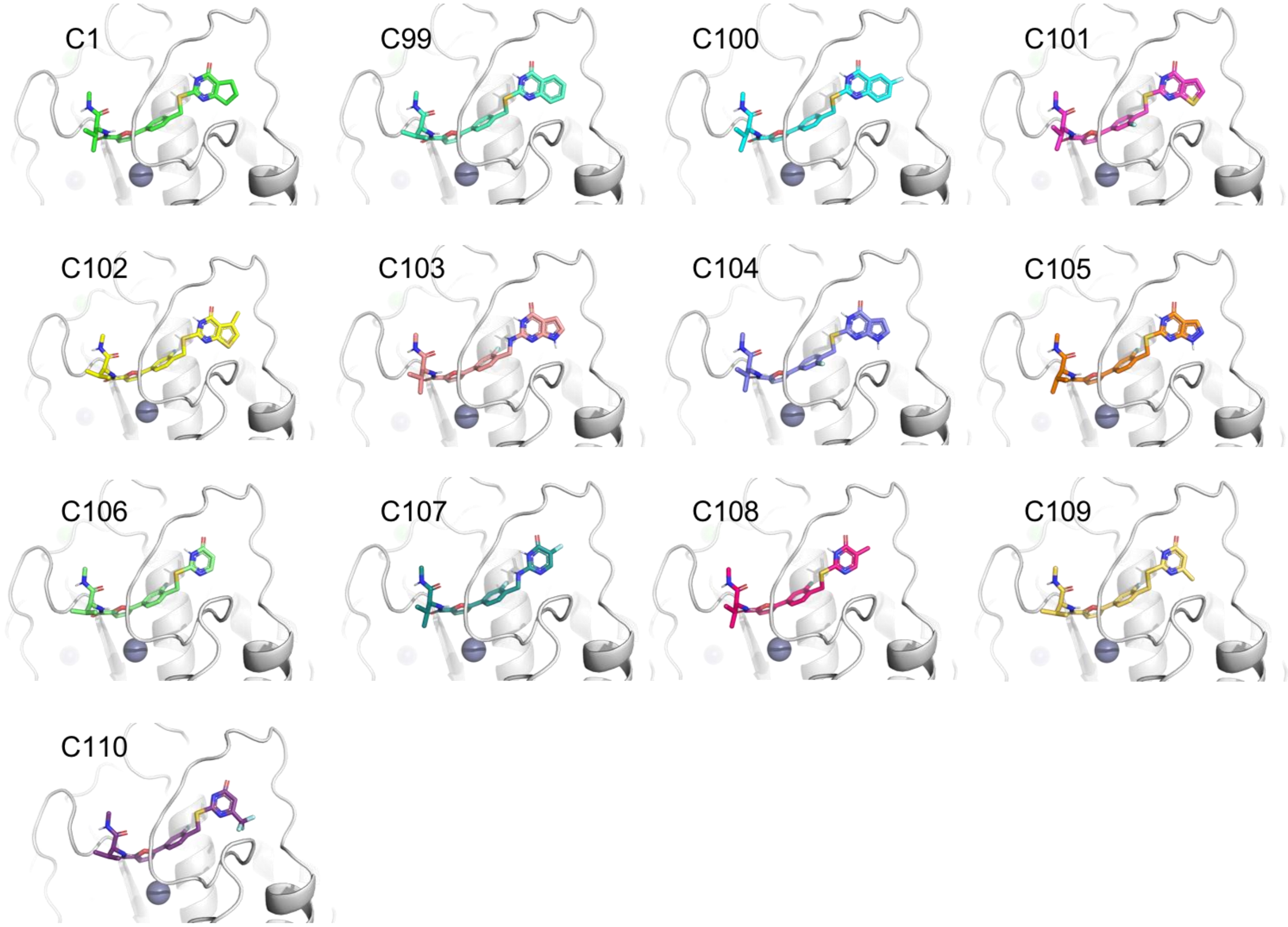

Figure S3. Glide docking results of C99-C110 to MMP-13: (A) The binding poses of all ligands are overlayed based on the binding pose of C1 in the X-ray co-crystal structure (PDB code: 5UWL); (B) The individual view of the bind poses of all ligands from docking studies.

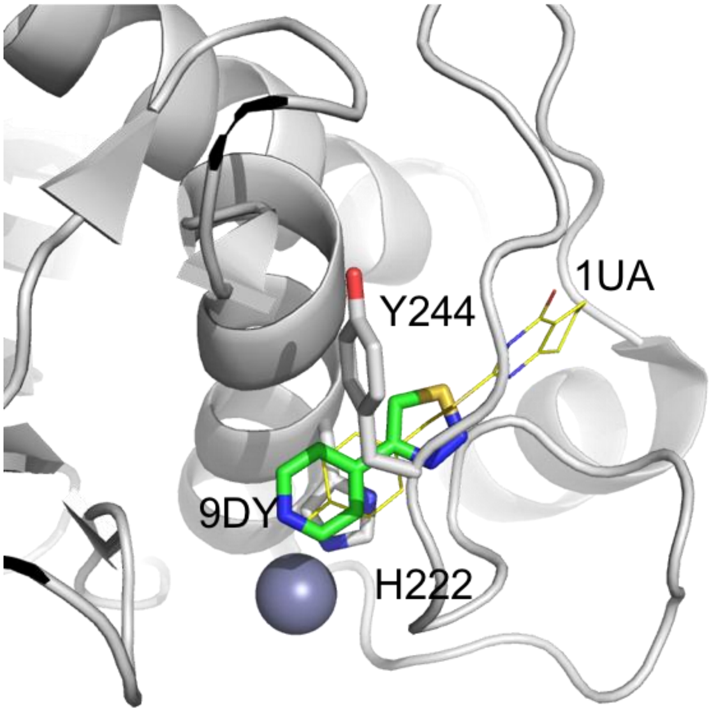

Figure S4. X-ray co-crystal structure of MMP-13 – 9DY (PDB code: 7JU8). The fragment (9DY, 4-(1,2,3-thiadiazole-4-yl)pyridine) is green. The imaginary 1UA structure is present as a yellow wire for comparison.

Table S1. The results of 10 independent MD simulations of MMP-13 – 1UA.

MD run	coordinate	Binding pose	$\Delta G_{binding}$ (N=20)[a]	$\Delta G_{MM/GBSA}$ (N=1000)[b]
1	PDB (4L19)	Pose1	-13.3 (±2.9)	-35.7 (±2.0)
2	PDB (4L19)	Pose2	-20.6 (±4.2)	-42.2 (±2.5)
3	5 ps equilibration in MD run1	Pose1	-14.6 (±3.5)	-35.1 (±2.1)
4	5 ps equilibration in MD run2	Open	-13.0 (±3.6)	-35.1 (±2.0)
5	10 ps equilibration in MD run1	Open	-14.9 (±5.3)	-35.0 (±2.0)
6	10 ps equilibration in MD run2	Pose2	-20.3 (±4.5)	-42.1 (±2.6)
7	15 ps equilibration in MD run1	Pose1	-12.7 (±5.1)	-35.5 (±2.0)
8	15 ps equilibration in MD run2	Open	-15.0 (±4.9)	-35.4 (±1.9)
9	20 ps equilibration in MD run1	Pose1	-12.6 (±3.8)	-35.5 (±1.9)
10	20 ps equilibration in MD run2	Open	-13.3 (±2.9)	-35.3 (±1.9)

[a] MM/GBSA calculation with the normal mode entropy approximation.

[b] MM/GBSA calculation without the entropy approximation

Table S2. Solute-solvent hydrogen bond analysis of C1 from the MD simulations of MMP-13 – C1 complex

Amino acid	count	Fractions
LIG@O3	21669	1.0835
LIG@O1	18040	0.9020
LIG@O4	13484	0.6742

Table S3. Water-bridged hydrogen bond analysis of C1 from the MD simulations of MMP-13 – C1 complex

Amino acid	Frames (total: 20,000)
G183	4046
L185	273
P242	95
Y244	883

Table S4. Structures of ligands used in MD simulations

Ligand code	Structure		Ligand code	Structure
1UA	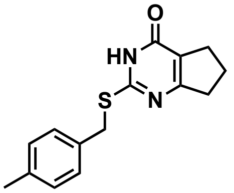		C104	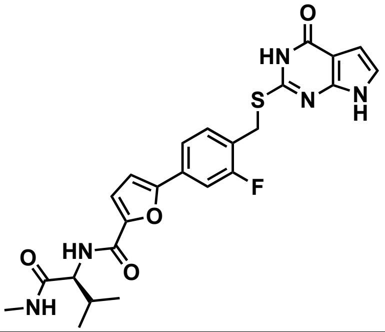
C1	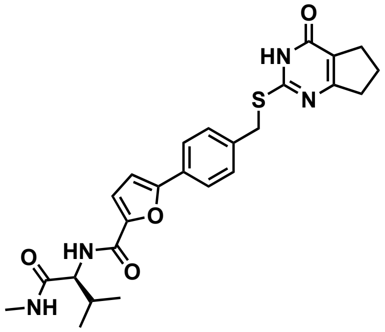		C105	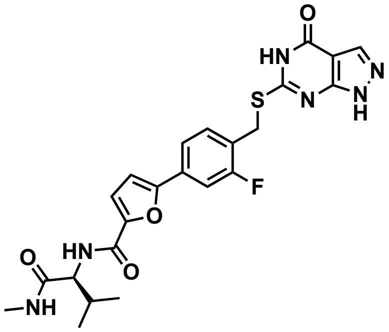
C2	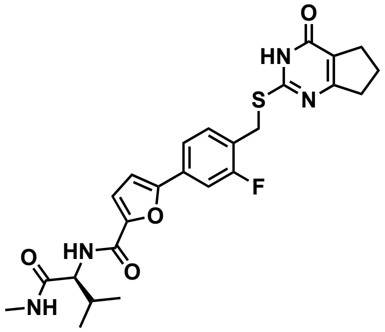		C106	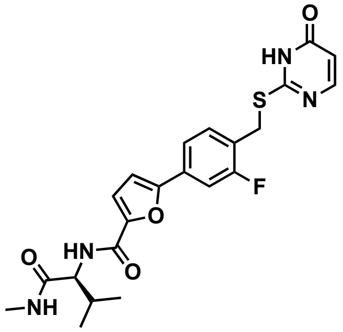
C99	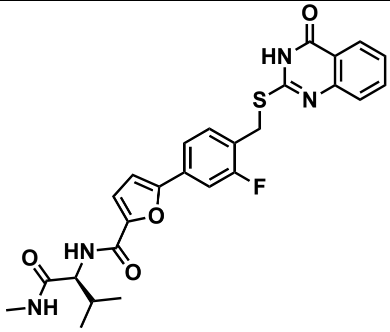		C107	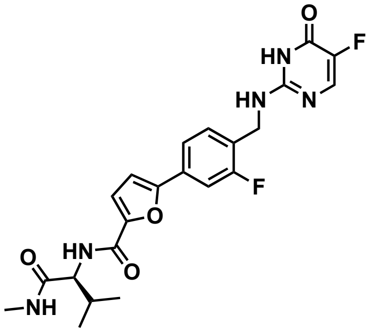

C100	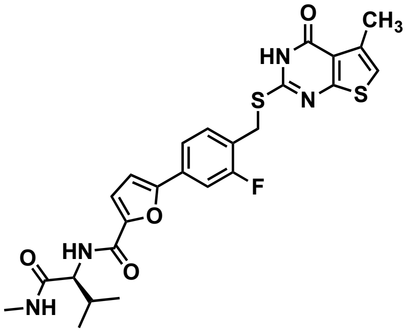		C108	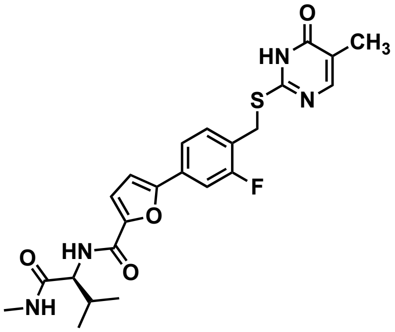
C101	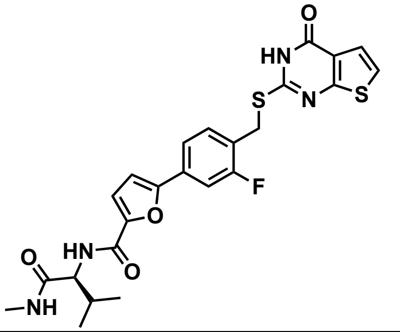		C109	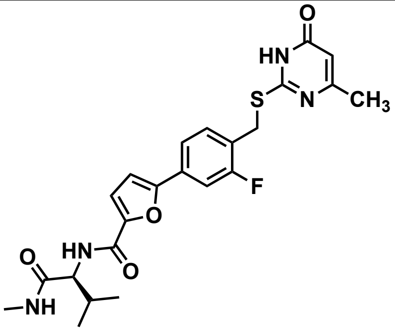
C102	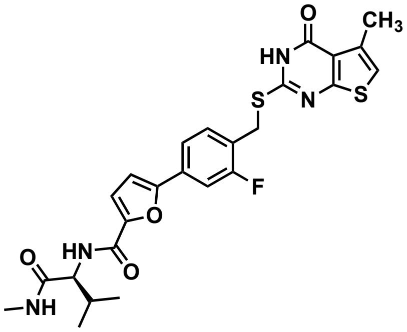		C110	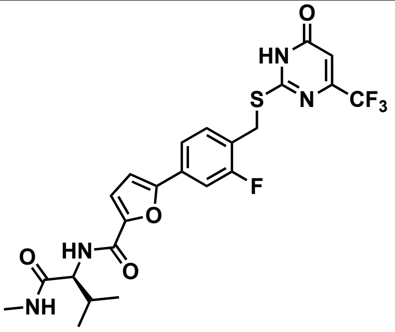
C103	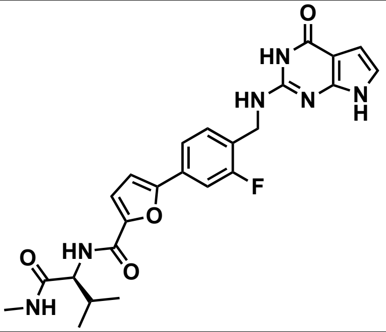			
